# Society Meetings

**Published:** 1914-11

**Authors:** 


### SOCIETY MEETINGS

---

**Brief reports and announcements of meetings of societies of Western New York, and those of general scope, are requested from Secretaries. Copy should be on hand the fifteenth of the month. Full transactions will be published at cost of composition.**

---

**The Buffalo Academy of Medicine** is holding its meetings for the season of 1914-15 at Orpheus Hall, 432 Franklin Street, Wednesday evenings, instead of Tuesday, as heretofore) at 8:30 p. m. Visiting physicians are always welcome and should secure an introduction to the Academy. Physicians of other places than Buffalo living within convenient distance should apply for non-resident membership.

Section of Surgery, October 7, Transfusion of Blood, A. H. Noehren; Insufflation Aether Anaesthesia, John Evans.

Section of Medicine, October 14, Eiweiss Milk as Used at

Finkelstein's Clinic, Douglas P. Arnold; Is Vision to be the Sole Intellectual Sense?—With Special Reference to Physical Diagnosis, A. L. Benedict.

Section of Obstetrics and Gynaecology, October 21, Twilight Sleep, Abraham J. Rongy, New York, discussion opened by P. W. Van Peyma.

---

**The Eighth District Branch of the Medical Society of the State of New York** held its ninth annual meeting at the Hotel Kaltenbach Annex, Niagara Falls, September 23 and 24. (Note: A full report of the meeting, expected, has failed to arrive in time for publication.)

---

**The Elmira Academy of Medicine** held its first meeting of season, in the Federation Building, October 7. Thrombosis of Mesentery, Complicating Puerperal Sepsis, W. A. De Witt of Blossburg, Pa.; Dr. A. Lande also presented a paper, title not announced.

---

**The Medical Society of the County of Monroe** held its regular meeting at the Hotel Seneca, Tuesday, October 14. Lewis H. Taylor of Wilkes-Barre read a paper on "The Medical Library: Its Value and Use to the Profession."

---

**The Rochester Academy of Medicine,** Section LL, Surgery, etc., met in the Eastman Building, University of Rochester, October 14. Anatomy, Embryology and Histology of the Ductless Glands, John M. Swan; Pituitary, David B. Jewett, Thyroid and Parathyroids, C. K. Haskell, Adrenal and Thymus, W. E. Bowen.

---

**The Medical Society of the County of Erie** held its regular meeting October 19, in the Lecture Hall of the University of Buffalo. The Role of the Genital Organs in Acute and Chronic Arthritis, Henry Adsit; Arthritis of the Joints of the Hand following Colles' Fracture, Prescott Le Breton.

---

**The Gross Medical Club** held their stated meeting at the residence of Dr. Clarence MacKay, Lancaster, Friday, October 16th, under the supervision of Dr. A. G. Bennett, President.

A very interesting didactic talk was given by Dr. Marshall Clinton on the diagnosis of abdominal tumors. The subject was thoroughly discussed by Drs. Jas. E. King and MacLeod who emphasized the value of the X-ray in the diagnosis of these growths.

A case of abscess of the litoris, a very rare condition was reported by Dr. Gipple of Alden.

Dr. King reported the case of a child, 3½ years old from whom he had removed a hair pin from the bladder, suprapubically.

The history of this case was most peculiar and amusing. Several children of about the same age were playing, when it was suggested an examination was necessary, a hair pin being used for the purpose, which was inserted into the urethra, slipped from the grasp and entered the bladder. A boy of about the same age proposed an operation for its extraction, and with a pair of scissors attempted the feat but was unsuccessful. Although it had been in the bladder two months no symptoms, except nocturnal enuresis was complained of. Examination for this condition revealed a hard substance in the bladder, suprapubic section was performed and the pin heavily coated with urinary salts was found. The surprising part is, there had been no pain at any time.

Dr. Byron E. Smith of Angola was elected to membership.

President Bennett appointed Drs. Jas. E. King, J. Henry Dowd and Albert E. Berry as a committee on membership.

---

The forty sixth annual meeting of **Central and Western New York Medical Association** was held at the Hotel Onandaga, Syracuse, September 30, 1914. There was a large attendance of physicians from the counties which compose the society. The paper of Dr. Albert J. Jochner of Chicago, on Thyroid Surgery was especially interesting; the author who has devoted a life-time to this subject, saying that not over 25% of Goitres should be operated upon; the other 75% being cured by rest, diet and medication. He warned the profession not to use Thyroid Extracts in the treatment of Goitre. He then gave a very elaborate description of his operation with lantern slides of each step, especially calling attention to the median thyroid artery which is often overlooked, severe hemorrhage resulting. Dr. Floyd S. Crego of Buffalo opened the discussion from the medical standpoint, commending Dr. Ochsner's conservatism as to operation. Dr. Crego said he often called in the surgeon to operate but had seen many cases recover with medicine. He especially recommended various forms of Calcium as it had been shown that a deficit of calcium was found in these cases. The treatment Dr. Crego recommended was Calcium, Digitalis and Anti-thymroidin Serum or Thyrodectine, rest and diet. He reported four severe cases recovered within a year. The members were entertained at luncheon by the Onondaga County Medical Society. After lunch the business meeting was held, at which the following officers were elected for the ensuing year: President, D. C. O. Boswell, Rochester, N. Y. First Vice-president, Dr. A. L. Benedict, Buffalo, N. Y. Second Vice-president, Dr. Hugh H. Carpenter, Oneida, N. Y. Secretary, Dr. John J. Buettner,

Syracuse, N. Y. Treasurer, Dr. Thos. Laurie, Auburn, N. Y. Meeting adjourned at 6 p. m. to meet next year in Rochester, N. Y. In the evening a banquet was participated in by many members in conjunction with the Sixth District Branch of the N. Y. State Medical Society. After the banquet a vaudeville entertainment was given. The meeting, entertainment and attendance were voted among the best the society has ever held, and a very bright future is before the society.

---

**The Association of Military Surgeons of the U. S.** held its 23d annual meeting in Cincinnati, September 29-October 2. An excellent program of scientific and executive papers was presented and ample entertainment was provided. The following officers were elected: President, Col. Jefferson R. Kean, U. S. A.; vice-presidents, Surgeon-Gen. Rupert Blue, U. S. P. H. S., Medical Inspector, Geo. A. Lung, U. S. N., Col. Henry Allers, Chief Surgeon of the N. J. Guard; secretary, Brigadier Gen. S. C. Stanton, Illinois Guard, ret.; treasurer, Maj. H. A. Arnold, Penn. Guard; asst. secretary, Asst. Surg. Gen. Wm. Colby Rucker, U. S. P. H. S. Lt. Col. A. H. Briggs was the sole attendant from Buffalo. The next meeting will be held in Washington. An earnest effort will be made to include in the membership every medical officer of the National Guard, not so much in the interest of the Association as a whole, as in that of the prospective members.

---

**The American Assn. for Study and Prevention of Infant Mortality** will hold its 5th annual meeting in Boston, November 12-14. Clinics and an exhibit will be arranged in addition to papers which will deal mainly with: prenatal care, need for increased and improved hospital service, institutional mortality and continuation schools of home-making. The dues for active members are $3, for affiliated societies $5. Checks should be made out to Austin McLanahan, Treas., 1211 Cathedral Street, Baltimore.

---

**The Rochester Medical Association — Hospital Medical Society,** announces the following program for the present year:

October 15—New Growth of Mediastinum, with Report of Two Cases. Frederick W. Seymour.

October 29—Blood Transfusion. Fred J. Garlick.

November 12—Bacillus Lactis Bulgaricus Inplantation during Intestinal Disease of Infants. William H. Cadmus.

November 27—The Cerebro-Spinal Fluid in Diagnosis. Charles S. Sutter.

December 10—Meeting with the President.

January 7—Brain Abscess of Otitic Origin. Bradford A. Richards.

January 21—Diseases and Injuries in and about the Hip Joint. Ralph R. Fitch.

February 4—Some Aspects of Surgery of the Colon. Walter A. Calihan.

February 18—Subject and Reader to be announced.

March 4—Observations on the Ductless Glands. Seelye W. Little.

March 18—Subject and Reader to be announced.

April 1—Erysipelas: Some Peculiar Manifestations. George C. Whitney.

April 15—Treatment of Retro-Displacement of the Uterus. George M. Gelser.

April 29—Subject and Reader to be announced.

May 13—The Annual Meeting.

Officers: President, Willis E. Bowen; vice-president, George M. Gelser; secretary and treasurer, Howard L. Prince.

Trustees: Richard M. Moore, Norris G. Orchard, James K. Quigley, Ralph R. Fitch, Clayton K. Haskell.

